# A case of xenon inhalation therapy for respiratory failure and neuropsychiatric disorders associated with COVID-19

**DOI:** 10.17179/excli2021-4316

**Published:** 2021-10-28

**Authors:** Vladimir Vasil'evich Udut, Sergei Alexandrovich Naumov, Diana Nikolaevna Evtushenko, Elena Vladimirovna Udut, Sergei Sergeevich Naumov, Gleb Nikolaevich Zyuz'kov

**Affiliations:** 1 Tomsk National Research Medical Center, Russian Academy of Sciences, Goldberg Research Institute of Pharmacology and Regenerative Medicine, Tomsk, Russia; 2 Tomsk State University, Tomsk, Russia; 3 Siberian State Medical University, Tomsk, Russia

**Keywords:** SARS-CoV-2, xenon, surfactant, respiratory failure, neuropsychiatric disorders, molecular modeling

## Abstract

Acute respiratory distress syndrome (ARDS) is the main danger to the life of patients with pneumonia caused by SARS-CoV-2. At the same time, respiratory failure (RF) after ARDS can persist for a long time despite intensive therapy. Therefore, it is important to develop new effective approaches for restoring the ventilation function of the lungs after COVID-19. Here, we present a case report of effective application of short-term inhalations of xenon-oxygen (Xe/O_2_) gas mixture for treatment of RF and neuropsychiatric disorders (NPD) associated with COVID-19. The patient inhaled a gas mixture of 70 % Xe and 30 % O_2_. We used multispiral computed tomography, evaluated psychometry, studied hematological and biochemical blood parameters, and applied some other methods of clinical studies to assess the therapeutic effectiveness of Xe inhalation. Also, we studied the mechanism of action of xenon with computer modeling. The clinical case showed the high efficacy of Xe/O_2_ mixture for treating severe RF and NPD after SARS-CoV-2 infection. Xenon inhalations dramatically increased oxygen saturation and the degree of pneumatization of the lungs. We found out that in coronavirus pneumonia, saturated phospholipids of surfactant are transferred to the solid-ordered phase, which disrupts the surface tension of the alveoli and alveolar gas exchange. Using molecular modeling methods, we demonstrated that the xenon atom increases the distance between the acyl chains of phospholipids due to the van der Waals dispersion interaction. These changes allow for the phase transition of phospholipids from the solid-ordered phase to the liquid phase and restore the functional activity of the surfactant. The findings suggest the feasibility of conducting studies on the effectiveness of Xe/O_2 _inhalations for treating ARDS in SARS-CoV-2 infection.

## Introduction

Despite the ongoing active vaccination against severe acute respiratory syndrome coronavirus 2 infection (SARS-CoV-2), the disease continues to pose a serious threat to the health of people around the world (Sreepadmanabh et al., 2020[33]; Alsharif and Qurashi, 2021[2]; Majumder, Minko, 2021[22]; Marian, 2021[23]). Acute respiratory distress syndrome (ARDS) is the main danger to the life of patients with pneumonia caused by SARS-CoV-2 (Lai et al., 2020[18]). At the same time, respiratory failure (RF) after ARDS can persist for a long time despite intensive therapy (Rogers, et al., 2020[27]; Biancari et al., 2021[6]; Garfield et al., 2021[13]).

An inflammatory damage to lung terminal parts plays the main role in the pathogenesis of their ventilation function disorders in coronavirus infection (Tay et al., 2020[35]). Lesions of alveolar and microcirculatory tissues are also accompanied by hemostatic disturbances (Colling and Kanthi, 2020[7]; Miesbach and Makris, 2020[25]), which is confirmed with various clinical research methods: typical computed tomography (CT) manifestations (peripheral, subpleural pulmonary ground-glass opacities, often in the lower lobes) (Pontone et al., 2021[26]); high D-dimer levels in the blood indicating compensatory enhancement of fibrinolysis due to activation of thrombosis (Rostami and Mansouritorghabeh, 2020[28]). 

Severe damage to the terminal parts of the lungs results in a lengthy lung function recovery in COVID-19: atelectasis formation, microvascular thrombosis, and disturbances of lung alveoli micromechanics (structure and function of surfactant and tissue components) (Zhao et al., 2020[39]; Tong et al., 2021[36]). These changes are often the reason for maintaining respiratory failure for a long time, which in turn significantly increases the risk of developing pulmonary fibrosis after SARS-CoV-2 infection (George et al., 2020[14]; Tale et al., 2020[34]).

Post-COVID-19 rehabilitation mainly consists in therapeutic respiratory gymnastics, physiotherapeutic procedures, drugs for lung function restoration (bronchodilators, anticoagulants antiplatelets, anti-inflammatory agents) (Asly and Hazim, 2020[3]; Demeco et al., 2020[8]). These approaches aim to restore lung functions. However, due to SARS-CoV-2 targeting of nerve tissue (Li et al., 2020[20]; Sepehrinezhad et al., 2020[30]) patients undergoing COVID-19 often develop neuropsychiatric disorders (NPD). They complain about depression, anxiety disorders, insomnia, etc. (Abboud et al., 2020[1]; Rogers et al., 2020[27]). However, pharmacotherapy for neuropsychiatric conditions in COVID-19 patients remains extremely rare.

The effectiveness of inhalations of the xenon-oxygen (Xe/O_2_) gas mixture in depression, anxiety, insomnia, and pain syndromes not related to SARS-CoV-2 infection is well-known (Dobrovolsky et al., 2017[10]; Frontera, 2020[12]; Maze and Laitio, 2020[24]; Shao et al., 2020[31]). In this regard, we suggested that short-term inhalations of the Xe/O_2_ mixture could be effective for treating complications caused by SARS-CoV-2.

Our research aimed to study the effectiveness of short-term inhalation of the Xe/O_2_ gas mixture for treating RF and NPD associated with SARS-CoV-2, as well as to identify the mechanisms underlying this therapy approach.

## Case Report

**Figure 1:**
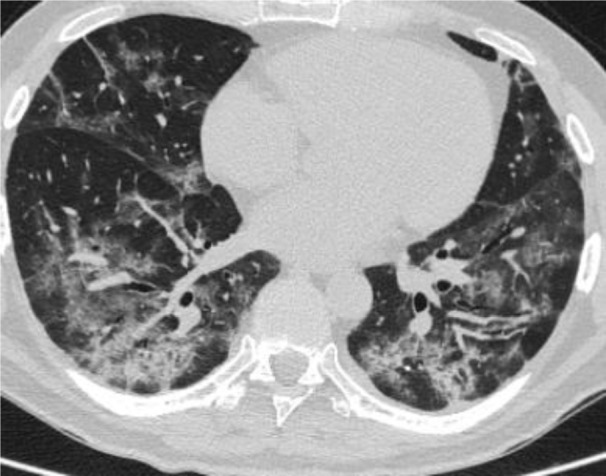
Chest MS CT scan after hospital discharge before xenon-oxygen gas mixture treatment

Patient A is 48 years old. There are complaints about the shortness of breath, weakness, chronic fatigue, dry cough, chest pain, anxiety, depressed mood. The medical history says that Patient A has been sick for 30 days. The patient stayed in the hospital for 17 days. The main discharge diagnosis: U 07.1 - COVID-19, SARS-CoV-2 virus (Novel Coronavirus (SARS-CoV-2) Fast Nucleic Acid Detection Kit (PCR-Fluorescence Probing, Jiangsu CoWin Biotech Co., Ltd., China). Complications: J12.8 - viral bilateral pneumonia, severe course; type 3 respiratory failure (the appearance of shortness of breath at rest). According to multispiral computed tomography (MS CT, Somatom Emotion 6 Slice Configuration, Siemens, Germany), ground-glass opacity is detected in 45 % of lung parenchyma (Figure 1(Fig. 1)).

The inhalations were performed once a day, in the morning. Each inhalation lasted one minute. Patient A inhaled a gas mixture of 70 % xenon (Xe) and 30 % oxygen (O_2)_ for 5 days. We used a xenon inhalation apparatus SAKI-stationary (LLC Scientific Corporation Biology Gas Service, Russia) and medical xenon XeMed (LLC Akela-N, Russia). The gas mixture from the apparatus was passed through a precision regulator at a pressure of 0.02 MPa (500 ml/min flow rate) into a 3,000 ml breathing bag. 

Before and after each Xe/O_2_ inhalation we determined respiratory rate (RR); heart rate (HR); blood oxygen saturation (SpO_2_) (Pulse Oxymeter Jziki pulsoximeter, China). During inhalations, the patient's condition was monitored according to subjective parameters (dizziness, euphoria, paresthesia) and objective parameters SpO_2_ level, RR, HR, nystagmus.

Before the start of treatment with the gas mixture and 11 days after the first inhalation, we examined MS CT hematological (hemoglobin, red blood cell count, platelet count, white blood cell count, leukocyte differential count, erythrocyte sedimentation rate (ESR)), and biochemical (D-dimer, alanine aminotransferase (ALT), aspartate aminotransferase (AST), total protein, glucose, cholesterol, total bilirubin, urea, creatinine, C-reactive protein) parameters and also investigated psychometry. Depression (according to the Hamilton Depression Rating Scale (HDRS)), anxiety (according to the Hamilton Anxiety Scale (HAS)) (Lu et al., 2020[21]), sleep disorders - sleepiness (according to the Epworth Daytime Sleepiness Scale (ESS) (Walker et al., 2020[38])), and insomnia (according to the Athens Scale (AIS) (Voitsidis et al., 2020[37])) were diagnosed.

Before starting the therapy the examination results were: RR at rest was 30 breaths per minute; HR was 94 beats per minute; SpO_2_ was 91 %. Psychometry data: moderate depression (18 points on the HDRS); moderate anxiety (22 points on the HAS); moderate excess daytime sleepiness (14 points on the ESS) and insomnia (2 points on the AIS). 

The short-term inhalation course of the Xe/O_2_ gas mixture was accompanied by a significant change in the patient's parameters (Table 1(Tab. 1)). After the first and second inhalation, a decrease in HR and RR was recorded, as well as a significant increase in SpO_2_. After the third therapy session, the studied indicators reached the norm. The SpO_2_ after each inhalation session increased significantly, and the RR after the fourth and fifth inhalations decreased.

The subjective examination showed that the patient's condition improved significantly after the first inhalation session. Patient A noted a decrease in chest pain, a change in the coughing (the cough became moist with a moderate sputum outflow), normalization of sleep, and a decrease in weakness. On the 7^th^ day of the therapy, the patient stopped feeling chest pain, shortness of breath; the cough became rare with a small amount of sputum. 

The psychometry evaluation on day 11 showed: mild depression (9 points on the HDRS), mild anxiety (14 points on the HAS), no daytime sleepiness (6 points on the ESS), and insomnia (1 point on the AIS).

**Figure 2:**
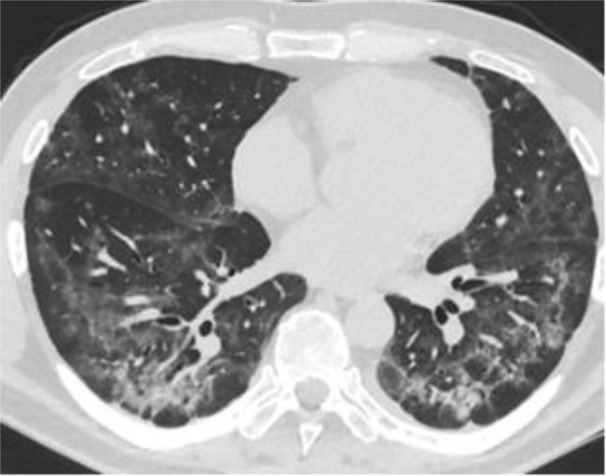
Chest MS CT scans after a course of inhalation with Xe/O_2_ gas mixture

The MS CT revealed a significant decrease in the foci of lung tissue damage: ground-glass opacity is detected in 15 % of lung parenchyma. There was an increase in pneumatization of the lungs (Figure 2(Fig. 2)).

Significant changes were also recorded in peripheral blood counts (Table 2(Tab. 2)). There was a significant decrease in the white blood cell count (more than 2 times) and normalization of the differential blood count. Meanwhile, a slight decrease in platelet count, red blood cells, and hemoglobin were observed. At the same time, ESR increased from 29 mm/h to 42 mm/h. But the change in this indicator cannot be considered as a criterion for the intensity of the inflammatory reaction, since a decrease in the content of C-reactive protein was recorded. In addition, decreased levels of D-dimer, transaminases (ALT and AST), and blood glucose were also revealed (Table 3(Tab. 3)).

## Discussion

The pandemic COVID-19 posed many challenges to the scientific medical community. First and foremost, specialists focused on the development of vaccines capable of inducing a reliable immune response in the body (Alsharif and Qurashi, 2021[2]; Majumder and Minko, 2021[22]; Marian, 2021[23]). However, the high genetic variability of SARS-CoV-2 (Badua et al., 2021[4]), the imperfection of vaccine immunity (Kang et al., 2020[16]), as well as many unknown factors in the pathogenesis of COVID-19 still hinder the achievement of an adequate level of population immunity (Lazarevic et al., 2021[19]). In this regard, developing novel approaches to the treatment of SARS-CoV-2 infection and its complications is an urgent task. Scientists and medicians are currently working to create new antiviral agents and repurpose conventional drugs (Marian, 2021[23]). 

Although approaches to the rehabilitation of various diseases with the help of some noble gases (helium, xenon, argon, krypton, etc.) are known (Frontera, 2020[12]; Kang et al., 2020[16]), researchers pay very little attention to medical gases that could be used as treatments for viral pneumonia. 

Given the permission in the Russian Federation for the use of the Xe/O_2_ gas mixture in medicine (Medical technology NoFS 2010/227 dated 17.06.2010. Method for the correction of acute and chronic stress disorders based on the inhalation of therapeutic doses of XeMed brand xenon). We were the first to attempt to treat SARS-CoV-2-associated respiratory failure and postcovid mental disorders with xenon.

The clinical case demonstrates the high efficacy of the Xe/O_2_ gas mixture for treating RF and NPD caused by SARS-CoV-2 infection. 26 COVID-19 have been treated with this approach so far. In all cases, a pronounced therapeutic effect was revealed. The study is not completed, the data is still being processed. 

The results of computer modeling of its mechanisms of action can provide a scientific and theoretical explanation of the revealed therapeutic effect of Xe in viral lung damage. The molecular modeling was carried out using the software package Gaussian09 (computational cluster SKIF Cyberia, Tomsk State University, Tomsk, Russia) by means of the density functional theory, hybrid functionality B3LYP and a basic set of lanl2dz functions parameterized for elements of 1-6 groups of the Mendeleev's Periodic System of Chemical Elements (Frisch et al., 2016[11]). The calculation results were visualized with the software package GaussView 5.0.8. (Dennington et al., 2009[9]).

**Figure 3:**
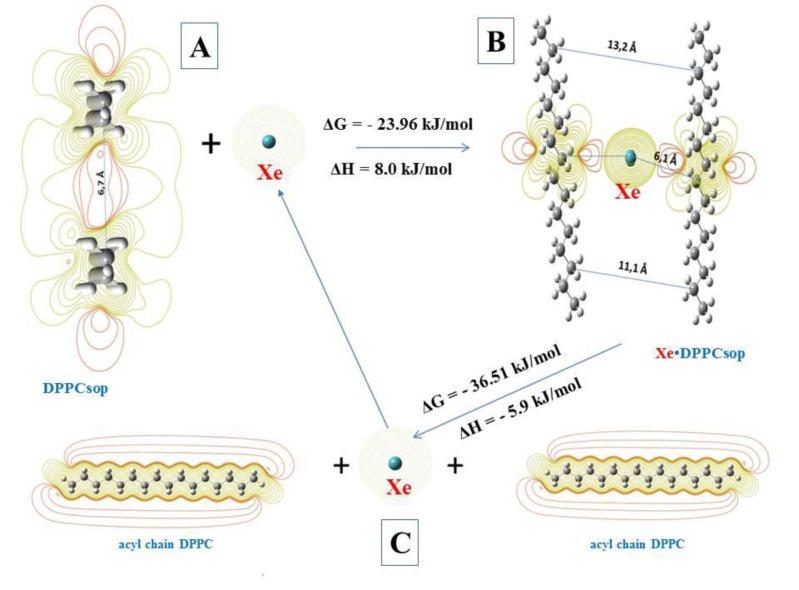
Processes of the interaction of xenon atom (Xe) with acyl chains of DPPC. A - Xe interaction with the bilayer of solid-ordered phase DPPC (DPPCsop); B - intermediate complex of Xe with two acyl chains DPPCsop (Xe∙ DPPCsop); C - decay of the intermediate complex to form individual acyl chains DPPC and free Xe

The surfactant plays an important role in the ventilation function of the lungs and ensures a proper level of gas exchange in them (Tong et al., 2021[36]). Surfactant phospholipids at the pulmonary air-liquid (blood) interface are formed by a monolayer providing a considerable reduction in surface tension in alveoli required for lung stabilization at the final exhalation phase (Bernhard, 2016[5]). Half of the surface-active phospholipids are known to consist of saturated species represented mainly by dipalmitoylphosphatidylcholine (DPPC) (Ghati et al., 2021[15]). The acyl chains of DPPC are residues of palmitic (hexadecanoic) acid. Therefore, molecules of unbranched long-chain C_16_H_34_ saturated hydrocarbons were taken for modeling the system. Also, a characteristic feature of aggregation of phospholipids and their transition to solid-ordered phase is a significant decrease in lateral mobility, which determines the reduction of surface tension (Bernhard, 2016[5]). As a result, in the solid-phase modeling system, the geometry of the surfactant was optimized. This was necessary to determine the minimum distance over which hydrocarbon molecules can be approached. In the course of computer modeling, it was determined that this distance is about 6.7 Å. The molecular electrostatic potential (MEP) distribution maps revealed the formation of a common region of negative MEP (red isoline) between hydrocarbon molecules (Figure 3A(Fig. 3)). This phenomenon explains the low lateral mobility of hydrocarbons in the solid-ordered phase.

It is known that a xenon atom has 54 electrons, which are easily polarized due to the Van der Waals dispersion interaction (Kleshchonok and Tkatchenko, 2018[17]). That is why the introduction of the Xe atom into the model system led to the emergence of a positive MEP region surrounded by two regions of negative MEP acyl (hydrocarbon) chains (Figure 3B(Fig. 3)).

Thus, the appearance of the xenon atom nearly doubled the distance between hydrocarbons. The change in Gibbs energy (ΔG) was -23.96 kJ/mol. Moreover, the enthalpy change (ΔH) was +8 kJ/mol. These findings indicate the possibility of spontaneous occurrence of the detected endothermic process (Song et al., 2020[32]).

In the second stage of the reaction (at ΔG = - 36.51 kJ/mol; ΔH = -5.9 kJ/mol) Xe is released and the acyl chains of phospholipids acquire an individual lateral mobility (due to the phase transition from the solid-ordered phase to the liquid phase). On the one hand, these changes are the basis for the restoration of the surfactant surface-active film. On the other hand, the released Xe atom immediately re-enters the cycle of interaction with the acyl chains of phospholipids in the solid-ordered phase. Moreover, the released xenon atom will interact with the acyl chains of phospholipids only of the solid-ordered phase of surfactant, but not with molecules of a normally functioning surfactant (because ΔG for such a process is greater than zero (Song et al., 2020[32])) (Figure 3C(Fig. 3)).

Thus, it is likely that it is the restoration of surface tension of the surfactant (Bernhard, 2016[5]; Ghati et al., 2021[15]) is the cause of improved lung ventilation function during xenon inhalation. The mechanisms of therapeutic action of this inert gas (Frontera, 2020[12]; Schiebler and Fain, 2020[29]) are based on its unique ability to polarize due to the Van der Waals dispersion interaction (Kleshchonok and Tkatchenko, 2018[17]). As a result of this, the surface-active film of surfactant is restored, which plays an important role in the functioning of alveoli (Bernhard, 2016[5]; Ghati et al., 2021[15]). The repeated cycle of the xenon interaction - its release after the reaction for further interaction - with the acyl chains of the solid-ordered phase of surfactant phospholipids is a mechanism that enables a prolonged effect of even short-term inhalations.

The findings suggest the feasibility of conducting studies on the effectiveness of Xe/O_2 _inhalations for treating ARDS in SARS-CoV-2 infection.

## Declaration

### Funding 

The studies were carried out within the framework of the state assignment of the Ministry of Science and Higher Education of Russia on topics No. 0550-2019-0011, No. 0550-2019-0012, and No. FGWM-2022-0018.

### Acknowledgments

We are grateful to Olga Nikolaevna Chumakova for her contribution to the data collection.

### Data availability statement

All data generated or analyzed during this study are included in this published article.

### Ethics approval 

The study was approved by the Institute's local Ethics Committee (Goldberg Research Institute of Pharmacology and Regenerative Medicine, Tomsk National Research Medical Center, Russian Academy of Sciences) (protocol No. GRIPh&RM-2020-01/07). 

### Consent to participate

Informed consent from the patient was obtained.

### Conflict of interests 

The authors declare no competing interests.

## Figures and Tables

**Table 1:**
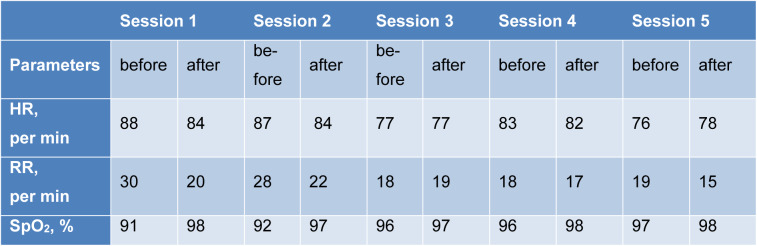
Dynamics of changes in HR, RR, SpO_2_, % during Xe/O_2_ gas mixture therapy

**Table 2:**
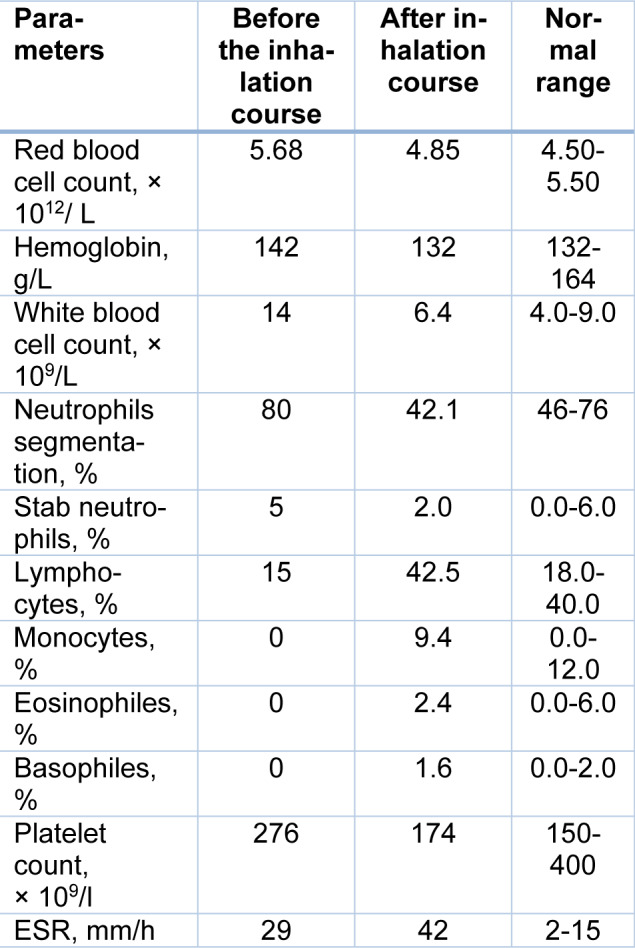
Hematological parameters

**Table 3:**
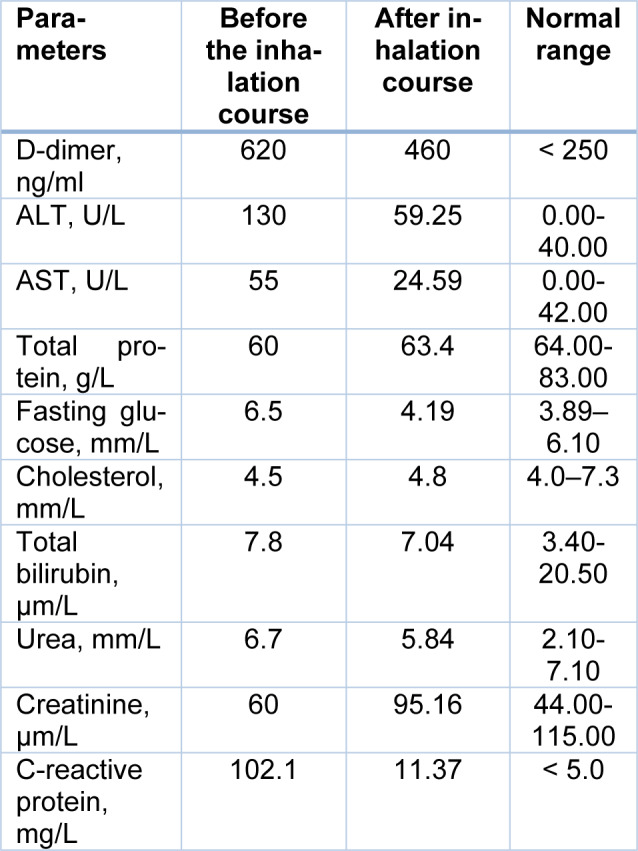
Biochemical parameters
